# Clinical outcome of intensity modulated radiotherapy for carcinoma showing thymus-like differentiation

**DOI:** 10.18632/oncotarget.11914

**Published:** 2016-09-08

**Authors:** Fangfang Kong, Hongmei Ying, Ruiping Zhai, Chengrun Du, Shuang Huang, Junjun Zhou, Xiayun He, Chaosu Hu, Zhuoying Wang, Tuanqi Sun, Qinghai Ji

**Affiliations:** ^1^ Department of Radiation Oncology, Fudan University Shanghai Cancer Center, Shanghai, P.R. China; ^2^ Department of Oncology, Shanghai Medical College, Fudan University, Shanghai, P.R. China; ^3^ Department of Radiation Oncology, Zhejiang Cancer Hospital, Hangzhou, P. R. China; ^4^ Department of Head and Neck Surgery, Fudan University Shanghai Cancer Center, Shanghai, P.R. China

**Keywords:** intensity-modulated radiotherapy, IMRT, radiotherapy, carcinoma showing thymus-like differentiation, CASTLE

## Abstract

**Purpose:**

To evaluate the efficacy and toxicity of adjuvant intensity-modulated radiotherapy (IMRT) after surgery for carcinoma showing thymus-like differentiation (CASTLE).

**Methods:**

Between September 2008 and June 2015, 14 CASTLE patients were retrospectively enrolled. The clinical features, treatment procedure and clinical outcomes were reviewed. All patients received postoperative IMRT. The radiation doses ranged from 56Gy/28 fractions to 66Gy/33 fractions. Treatment-related toxicities were graded by National Cancer Institute Common Toxicity Criteria (NCI-CTC) version 3.0.

**Results:**

After a median follow-up period of 42 months, only one patient suffered local recurrence and distant metastasis. The most frequently seen acute toxicities were mucositis and dermatitis (grade 1-2). No grade 3-4 toxicities were observed.

**Conclusions:**

Although based upon a small series of consecutively treated patients, our study showed that adjuvant IMRT provides satisfactory local-regional control for CASTLE, with acceptable toxicities. Further studies are still warranted to clarify our findings.

## INTRODUCTION

Carcinoma showing thymus-like differentiation (CASTLE) is a rare malignant tumor of the thyroid or adjacent soft tissue in the neck [1]. It has been postulated to arise from ectopic thymus or branchial pouch remnants [2]. Miyauchi et al [3] firstly described it as “Intrathyroidal epithelial thymoma (ITET)” in 1985. Chan and Rosai [2] later denominated it as “CASTLE” in 1991. It was designated as an independent clinicopathological entity of thyroid tumors by the World Health Organization (WHO) in 2004 [4].

There is no consensus on the management of CASTLE due to its rarity. Generally, curative surgery seems to be the best therapeutic option. However, CASTLE often invades adjacent soft tissue and causes lymph node metastasis. It was reported [5, 6] that lymph node metastasis happened in about 50% of the patients. And about 60% of the patients suffered tumor invasion to adjacent organs like the recurrent laryngeal nerve, the trachea, the esophagus and Jugular vein. As a result, adjuvant radiotherapy (RT) seems to be essential for the management of CASTLE [7]. It's reported that postoperative RT reduced the recurrence rate from 100% to 57% for patients with positive node [8].

Intensity-modulated radiotherapy (IMRT) is a major breakthrough of radiation technique in the past decade. The major advantage of IMRT is its capability of delivering high radiation dose to the tumor while sparing the adjacent normal organs [9-12]. Recently, IMRT has gained popularity in the treatment of head-and- neck cancer [13]. To our knowledge, information about adjuvant IMRT for CASTLE has not been reported up to now. Thus, we reviewed our clinical experience in 14 patients, focusing on the effectiveness and toxicities.

## RESULTS

### Patient characteristics and clinicopathological findings

The clinical characteristics of 14 patients are summarized in Table 1, 2, 3. There were six women and eight men. The median age was 48 years (range 38-60 years). The main first symptoms were painless, slow-growing neck mass in 11 patients, hoarseness in 1 patient and dysphagia in the other 1 patient. None of the patients complained of dyspnea. Seven patients had lymph node metastasis. Tumor extension to adjacent organs was found in 9 patients. The common sites for tumor extending included the recurrent laryngeal nerve (3/14, 21.4%), muscles (3/14, 21.4%), esophagus (2/14, 14.3%), carotid artery (2/14, 14.3%) and trachea (1/14, 7.1%).

As to immunohistochemistry, the tumor cells were positively immunoreactive for CD5 in 10 (71.4%) patients, partial positively in 2 (14.3%) patients and weakly positively in 2 (14.3%) patients. The expression of CD117 was analyzed in 11 patients, 7 (63.6%) of who were positive, 2 (18.2%) were partial positive, 1 (9.1%) was weakly positive and 1 (9.1%) was negative. The expression of P63 was analyzed in 12 patients, 11 (91.7%) of who were positive and 1 (8.3%) were partial positive. The expression of TTF-1 was analyzed in 11 patients, 9 (81.8%) of whom were negative and 2 (18.2%) were focally positive.

**Table 1:** Patient characteristics

	No. (%) of patients
Total	14
Median age (range)	48 (38-60)
Gender	
Male	8 (57.1)
Female	6 (42.9)
First symptom	
Neck mass	11 (78.6)
Dysphagia	1 (7.1)
Hoarseness	1 (7.1)
Unknown	1 (7.1)
Tumor location	
Left lobe	8 (57.1)
Right lobe	6 (42.9)
Tumor size (cm)	
≤2	1 (7.1)
2-4	8 (57.1)
>4	3 (21.4)
Unknown	2 (14.3)

**Table 2:** Lymph node metastasis and tumor extension

	No. (%) of patients
Lymph node metastasis (*n* = 14)	
Present	7 (50.0)
Absent	6 (42.9)
Unknown	1 (7.1)
Location of Lymph node (*n* = 7)	
Lateral	2 (28.6)
Central	5 (71.4)
No. of metastatic nodes (*n* = 14)	
≥3	2 (14.3)
1-2	5 (35.7)
0	6 (42.9)
Unknown	1 (7.1)
Tumor extension (*n* = 14)	
Present	9 (64.3)
Absent	3 (21.4)
Unknown	2 (14.3)
STE (n=14)	
RLN	3 (21.4)
Muscles	3 (21.4)
Esophagus	2 (14.3)
Carotid artery	2 (14.3)
Trachea	1 (7.1)
Jugular vein	1 (7.1)
Thymus	1 (7.1)
Parathyroid gland	1 (7.1)

Abbreviations: STE, site of tumor extension; RLN, recurrent laryngeal nerve.

**Table 3:** Treatment procedures and clinical outcome (*n = 14)*

	No. (%) of patients
Thyroidectomy	
Total	2 (14.3)
Subtotal	1 (7.1)
Lobectomy	7 (50.0)
Excision of the tumor	2 (14.3)
Palliative resection	2 (14.3)
Lymph node dissection	
Central node dissection	6 (42.9)
Not done	7 (50.0)
Unknown	1 (7.1)
CCRT	1 (7.1)
IMRT (Gy)	
66	4 (28.6)
60	9 (64.3)
56	1 (7.1)
IMRT duration (days)	
Median (range)	43 (39-67)
Median follow-up (months)	42 (7-92)
LR	
Present	1 (7.1)
Absent	13 (92.9)
DM	
Present	1 (7.1)
Absent	13 (92.9)

Abbreviations: CCRT, concurrent chemoradiotherapy; IMRT, intensity-modulated radiotherapy; LR, local recurrence; DM, distant metastasis.

### Dosimetric data for IMRT

Table 4 shows the dose-volume histogram (DVH) statistics for the target volume. All IMRT plans provided excellent coverage of the target volume. Only 0.3% of the gross tumor volume (GTV) and 1.6% of the clinical tumor volume (CTV) received less than 95% of the prescribed dose. The average maximum, mean and minimum dose delivered to GTV and CTV was 71.4, 68, 59.4 and 66.9, 62.4, 41.1Gy respectively.

**Table 4:** Dose-volume histograms (DVHs) statistics for fourteen patients with CASTLE

	GTV Average (range)	CTV Average (range)
Volume (cc)	149.4 (110.7-204.2)	221.8 (99.1-582.9)
Maximum dose (Gy)	71.4 (70.5-72.5)	66.9 (63.5-72.5)
Mean dose (Gy)	68.0 (67.4-68.8)	62.4 (57.7-66.8)
Minimum dose (Gy)	59.4 (57.9-62.0)	41.1 (7.8-55.5)
% volume receiving <95% of the prescribed dose	0.3 (0-0.7)	1.6 (0.1-12.0)
% volume receiving ≥100% of the prescribed dose	93.4 (86.9-97.7)	93.2 (77.6-99.0)
% volume receiving≥110% of the prescribed dose	0	14.3 (0-67.1)

Abbreviations: CASTLE, carcinoma showing thymus-like differentiation; GTV, gross tumor volume; CTV, clinical tumor volume.

### Toxicities

Acute toxicities related to IMRT by site and grades were listed in Table 5. The most frequently seen acute toxicities were mucositis and dermatitis (grade 1-2). Eleven patients (76.9%) suffered grade 1 skin pigmentation. No grade 3-4 toxicities were observed. The acute toxicities were well tolerated and all patients completed radiotherapy as planned. We made no attempt to report any late complications due to the relatively short follow-up period and small sample.

**Table 5:** Acute toxicities during IMRT

Toxicities	No. of patients by toxicity grade (%)				
	Grade 1	Grade 2	Grade 3	Grade 4	Grade 5
Dermatitis	10 (71.4)	4 (28.6)	0	0	0
Mucositis	4 (28.6)	10 (71.4)	0	0	0
Skin pigmentation	11 (78.6)	0	0	0	0

Abbreviation: IMRT, intensity-modulated radiotherapy.

### Survival outcome

The median follow-up period was 42 months (range 7-92 months). One patient suffered local recurrence and pulmonary metastasis simultaneously 37 months after surgery. After salvage surgery to the neck and pulmonary, this patient was alive with no evidence of disease at the last follow-up. No recurrence or metastasis was observed in the remaining patients.

**Figure 1:**
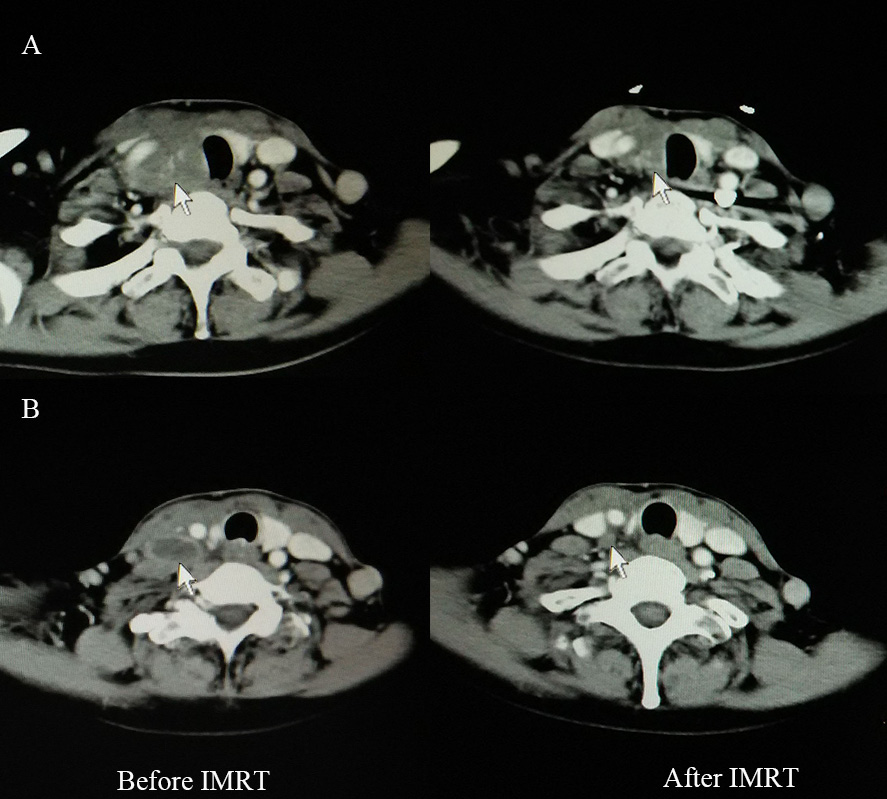
Computed tomography findings before (left) and after (right) IMRT for one patient with CASTLE who received palliative resection **A.** Disease extent for primary tumor (white arrow). **B.** Disease extent for involved lymph node (white arrow). IMRT, intensity-modulated radiotherapy; CASTLE, carcinoma showing thymus-like differentiation.

## DISCUSSION

CASTLE is a rare malignant of the thyroid. It accounts for only 0.1-0.15% of all thyroid cancers [14-16]. Up to now, about 80 cases have been reported in the English language literature, most of which are case reports. This is the first study about IMRT combined with surgery for the management of CASTLE.

It is reported that CASTLE occurs more frequently in females than males (female to male ratio, 1.3:1) [6, 17]. However, the sexual predominance seems to be opposite in China. Zhen et al [18] and Sun et al [15] reported a female to male ratio of 1:1.7 and 1:1.3 respectively. In the present series, the female to male ratio was 1:1.3. This may suggest the racial differences, though further study with a larger sample size is needed.

The clinical features of CASTLE are difficult to diagnose. Ito et al [6] investigated clinical features of 25 cases of CASTLE. And they found that the tumor enlarged slowly and often located in the lower part of the thyroid. It is lobulated and lack of calcification by ultrasonography. As to biomarkers, various markers including CD5, CEA, TTF-1, P63, cytokeratin, thyroglobulin and calcitonin were studied. However, CD5 seems to be the only potential marker [17, 19, 20]. The positive expression rates of CD5 in the literature ranged from 75% to 100% [6, 15, 18, 21, 22]. In the present study, we found that 85.7% of the cases expressed CD5 totally or partially. Instead, 9 of 11 (81.8%) cases were negatively expressed for TTF-1, which was the marker of thyroid follicular cells. It's worthwhile to note that negative expression of CD5 does not completely rule out CASTLE. The hematoxylin and eosin stained sections findings are the main basis for final diagnosis.

Optimal treatment modality is still uncertain for CASTLE due to its rarity. Some studies have showed that adjuvant RT could improve local control [3, 6-8, 16, 17, 23]. Choi et al [8] found that postoperative RT reduced the recurrence rate from 100% to 57% for patients with positive lymph node. In the study by Ito et al [6], among 10 patients who received adjuvant RT, no locoregional recurrence was observed, while 3 locoregional recurrences were observed in those who received surgery only. In the present study, all patients received adjuvant IMRT, only one patient suffered local recurrence. It is worth noting that the recurrence tumor was in the contralateral neck and outside the radiation field. Although the number of patients is modest, our study showed satisfactory local-regional control of adjuvant IMRT for CASTLE.

Two patients received palliative resection because of tumor invasion to the adjacent great vessels. IMRT was administered at a radical dose of 66 Gy in 33 fractions to them. One cycle of chemotherapy (paclitaxel 135mg/m^2^, day 1, cisplatin 25mg/m^2^/d, days1-3) was given to one of the patients concurrently with IMRT. Complete regression (CR) of the lymph node and partial regression (PR) of the primary tumor was obtained after IMRT (Figure 1). Both patients were alive with stable disease (SD) at the time of last follow-up. Tsutsui et al [5] reported a similar case in 2013. After radiotherapy, CR of the tumor was achieved and CT findings did not show local recurrence for about 7 years. In the study by Chow et al [16], radiotherapy combined with chemotherapy rendered the inoperable disease operable for one patient with locally advanced disease. These therefore remind us that curative IMRT combined with or without chemotherapy seems to be an alternative for patients with unresectable disease.

In conclusion, adjuvant IMRT provided satisfactory local-regional control for CASTLE, with acceptable toxicities. For patients with unresectable disease, curative IMRT combined with or without chemotherapy seems to be an effective choice. Further studies are still warranted to define optimal treatment modality.

## MATERIALS AND METHODS

### Patients

Fourteen patients with pathologically diagnosed CASTLE who were treated by postoperative IMRT in our institution between April 2008 and June 2015 were enrolled in this study. The clinicopathological data, therapeutic procedures, and clinical outcomes were retrospectively reviewed. All patients received surgical resection including total thyroidectomy (*n* = 2), subtotal thyroidectomy (*n* = 1), lobectomy (*n* = 7) and tumor excision (*n* = 2). Two patients received palliative resection because of the extended tumor invasion to adjacent great vessels. The workup before IMRT included a complete history and physical examination, blood tests, chest computed tomography (CT) or radiography, neck CT or Magnetic Resonance Imaging (MRI), abdominal ultrasound, and electrocardiogram.

### Intensity-modulate radiotherapy

IMRT started 1 to 2 months after surgery. Patients were immobilized in a thermoplastic mask of head and shoulder. Intravenous contrast-enhanced CT with a slice of 5mm from the head to the upper chest was performed for the delineation of the target volume. The target volumes were defined in accordance with the International Commission on Radiation Units and Measurements Reports 50 and 62. The gross tumor volume (GTV) included all primary gross tumors and involved lymph nodes determined by imaging and clinical findings. The clinical target volume (CTV) was defined as the GTV plus 5 to 10mm margin. For patients without residual disease, CTV was defined as the tumor bed plus 5mm margin to encompass any microscopic extension. The planning target volume (PTV) was expanded from the CTV by a margin of 3 to 5mm. Conventional fractionation (2.0Gy/day) was used. The prescribed dose delivered to patients without residual disease was 56 -60Gy in 28-30 fractions. For patients with residual disease, a radical dose of 66Gy in 33 fractions was delivered.

The organs at risk (OAR) included the spinal cord, parotid glands, larynx, trachea and lung. A 5 mm margin was added to the spinal cord during optimization to form the planning organ-at-risk volume (PRV).

### Patient evaluation

Patients were evaluated weekly for treatment response and toxicities during IMRT. After treatment, patients were follow-up every 3 months in the first 2 years, every 6 months from the third to the fifth year, and annually thereafter. Each follow-up after treatment included examination of the neck, thyroid function tests and ultrasound for the neck. Chest CT scan and ultrasound of abdomen were performed 3 months after IMRT and every 6-12 months thereafter.

Treatment-related toxicities were graded by National Cancer Institute Common Toxicity Criteria (NCI-CTC) version 3.0.
